# Early onset neutropenia in rituximab treated rheumatoid arthritis patients: a case series

**DOI:** 10.1093/rap/rkaf105

**Published:** 2025-09-14

**Authors:** Chung Mun Alice Lin, Alice R Lorenzi, John D Isaacs, Faye A H Cooles

**Affiliations:** Translational and Clinical Research Institute, Newcastle University, Newcastle-upon-Tyne, UK; Musculoskeletal Services, Newcastle upon Tyne Hospitals NHS Foundation Trust, Newcastle-upon-Tyne, UK; The NIHR Newcastle Biomedical Research Centre (BRC) based at the Newcastle Hospitals NHS Foundation Trust and Newcastle University, Newcastle-upon-Tyne, UK; Musculoskeletal Services, Newcastle upon Tyne Hospitals NHS Foundation Trust, Newcastle-upon-Tyne, UK; Translational and Clinical Research Institute, Newcastle University, Newcastle-upon-Tyne, UK; Musculoskeletal Services, Newcastle upon Tyne Hospitals NHS Foundation Trust, Newcastle-upon-Tyne, UK; The NIHR Newcastle Biomedical Research Centre (BRC) based at the Newcastle Hospitals NHS Foundation Trust and Newcastle University, Newcastle-upon-Tyne, UK; Translational and Clinical Research Institute, Newcastle University, Newcastle-upon-Tyne, UK; Musculoskeletal Services, Newcastle upon Tyne Hospitals NHS Foundation Trust, Newcastle-upon-Tyne, UK; The NIHR Newcastle Biomedical Research Centre (BRC) based at the Newcastle Hospitals NHS Foundation Trust and Newcastle University, Newcastle-upon-Tyne, UK

Key message

- Early monitoring of neutrophils in patients with rheumatoid arthritis post-rituximab may identify important drug-related complications.

Dear Editor, Rituximab is a chimeric monoclonal anti-CD20 antibody, used widely to treat multiple rheumatic diseases. It has a favourable safety profile; however, early-onset neutropenia (EON), defined as a neutrophil count of <2.0 × 10^9^/l within 4 weeks of treatment [1–2], is a rare complication. This unusual side effect has been previously described in SLE following rituximab but has not been reported in RA until now [1–2]. We present the first dedicated case series of EON in RA post-rituximab therapy. Rituximab treatment cycles for all patients were delivered 6-monthly and consisted of two pulses of 1 g rituximab with 100 mg methylprednisolone, given 2 weeks apart.

## Case 1

Sixty-seven-year-old female with anti-CCP, RF positive RA (diagnosed 2004) and asymptomatic primary biliary cirrhosis (AMA positive, ANA negative). Previous DMARDs included methotrexate, leflunomide, hydroxychloroquine, sulphasalazine and Adalimumab. She had periodic neutropenia (range 1.55–1.73 × 10^9^/l) on high-dose methotrexate and leflunomide, which resolved upon discontinuation. Rituximab was given in combination with oral methotrexate (7.5 mg/week), and 11 days after her third cycle, neutrophils fell from 1.85 × 10^9^/l to 0.25 × 10^9^/l. Blood film was normal, and she was given granulocyte colony-stimulating factor (G-CSF), with neutropenia resolving after 3 days. Following her fourth uneventful cycle of rituximab, she remained in clinical remission.

## Case 2

Seventy-one-year-old female with anti-CCP and RF positive RA (diagnosed 1985) and subacute cutaneous lupus (ANA, Ro-60 and La positive, C3 1.08 g/l, C4 0.2 g/l). The latter was diagnosed following development of an annular, photosensitive rash that had biopsy appearances of a lichenoid dermatitis and resolved with Dermovate ointment. She had no clinical features of SLE. Previous DMARDs included methotrexate, leflunomide and etanercept. Subcutaneous methotrexate (10 mg/week) was given in combination with Rituximab for 1 cycle, but later stopped due to nausea. Leflunomide was then trialled but quickly stopped due to a flare of her cutaneous lupus. Hydroxychloroquine (200 mg/day) was then commenced from the second Rituximab cycle. During her fourth cycle, neutrophils fell from 3.51 × 10^9^/l to 1.17 × 10^9^/l after 14 days. Blood film was normal, and neutropenia resolved spontaneously thirteen days later. She remains on rituximab with no further complications.

## Case 3

Thirty-five-year-old male with anti-CCP and RF positive RA (diagnosed 2012), ANA negative and asthma. Previous DMARDs included methotrexate, hydroxychloroquine, sulphasalazine and Etanercept. He had a single documented neutropenic (1.20 × 10^9^/l) episode when first initiated on methotrexate, but has since had normal neutrophils on a stable dose. Rituximab was given in combination with subcutaneous methotrexate (15 mg/week), and 14 days after his first cycle, neutrophils fell from 4.28 × 10^9^/l to 0.81 × 10^9^/l. Blood film was normal, and the neutropenia spontaneously resolved after 4 days. He remained on rituximab but had further EON following the first dose of his second cycle, with neutrophils falling from 3.89 × 10^9^/l to 0.18 × 10^9^/l 14 days after treatment. As previously, his neutrophil count spontaneously rose to 1.83 × 10^9^/l 5 days later. He has since had 4 further cycles with no reported complications.

## Discussion

This is the first dedicated case series to report EON following rituximab in RA (summarized in Table 1). We observed EON 11–14 days post-treatment, with all cases resolving either spontaneously or with G-CSF. This mirrors previously reported EON timeframes (5–28 days) as well as clinical trajectories [1–4]. Interestingly, the rituximab cycle number was irrelevant, with two patients developing EON after previous uneventful cycles. This was also seen in patients with lupus, neuromyelitis optica and bullous pemphigoid [1–5]. Felty’s syndrome was considered as a differential for all patients, but was excluded given the absence of splenomegaly.

**Table 1.** Summary of baseline demographics, treatment history, laboratory values and management

	Patient 1		Patient 2		Patient 3	
**(A) Baseline demographics**						
**Age**	67		71		35	
**Sex**	Female		Female		Male	
**Date of RA diagnosis**	2004		1985		2012	
**ACPA status**	Positive		Positive		Positive	
**RF status**	Positive		Positive		Positive	
**ANA status**	Negative		Positive (1:640)		Negative	
**Comorbidities**	Asymptomatic primary biliary cirrhosis (AMA positive)		Subacute cutaneous lupus (ANA, Ro-60 and La positive)		Asthma	
**(B) Treatment history**						
**Previous csDMARDs**	MTX, LFU, HCQ, SSZ		MTX, LFU		MTX, HCQ, SSZ	
**History of neutropenia on csDMARDs**	Yes—MTX and LFU		No		Yes—MTX	
**Previous bDMARDs**	Adalimumab		Etanercept		Etanercept	
**Date bDMARDs stopped**	January 2014		November 2017		May 2019	
**Baseline DAS-28 score pre-RTX**	3.06		5.57		3.86	
**Reason for switch to RTX**	Loss of efficacy with Adalimumab after 1 year		Adverse effects on Etanercept (skin rash)		Lack of effect on Etanercept (taken alongside MTX and SSZ)	
**Date RTX started**	June 2014		January 2018		June 2019	
**Additional therapy with RTX**	PO MTX (7.5 mg/week), stopped July 2015 following EON at third cycle		SC MTX (10 mg/week), stopped May 2018 due to nausea LFU (10 mg OD), stopped June 2018 due to a flare of cutaneous lupus HCQ (200 mg OD), commenced after second cycle (to manage cutaneous lupus)		SC MTX (15 mg/week) continued alongside Rituximab	
**Total number of RTX cycles**	4 (stopped after fourth due to drug-free remission with DAS 0.67).		13 to date and ongoing with Rituximab spaced out to 9-monthly after the eighth cycle.		6 (stopped September 2021 and switched to Abatacept in Feb 2022)	
**Cycle of RTX when EON developed**	Third cycle		Fourth cycle		First cycle	
**(C) Laboratory Values**						
	**Pre-RTX**	**Post-RTX**	**Pre-RTX**	**Post-RTX**	**Pre-RTX**	**Post-RTX**

**Hb (g/l)**	123	120	133	136	140	141
**Plts (×10^9^/l)**	189	208	343	346	218	253
**WCC (×10^9^/l)**	3.19	1.89	5.46	2.79	6.62	2.49
**Neutrophils (×10^9^/l)**	***1.85***	***0.25***	***3.51***	***1.17***	***4.28***	***0.81***
**Lymphocytes (×10^9^/l)**	0.85	1.21	1.14	1.12	1.67	1.12
**Monocytes (×10^9^/l)**	0.36	0.35	0.55	0.38	0.65	0.52
**Eosinophils (×10^9^/l)**	0.12	0.06	0.21	0.09	0.01	0.02
**Basophils (×10^9^/l)**	0.01	0.02	0.05	0.03	0.01	0.02
**(D) Management of EON**						
**Time until EON developed**	11 days		14 days		14 days	
**Blood film**	Normal		Normal		Normal	
**Treatment for EON**	G-CSF		Spontaneous resolution		Spontaneous resolution	
**Time until EON resolved**	3 days		13 days		4 days	
**Further EON on subsequent RTX cycles**	No		No		Yes—during second cycle only	

(A) Summary table of baseline patient demographics. (B) Treatment history and timeline. (C) Haematology laboratory values pre- versus post-Rituximab treatment. (D) Management of the neutropenia post-rituximab therapy. bDMARDs, biologic disease-modifying anti-rheumatic drugs; csDMARDs, conventional synthetic disease-modifying anti-rheumatic drugs; ACPA, Anti-citrullinated protein antibody; AMA. anti-mitochondrial antibody; ANA. anti-nuclear antibody; DAS, disease activity score; EON, early-onset neutropenia; G-CSF, granulocyte colony-stimulating factor; Hb, haemoglobin; HCQ, hydroxychloroquine; LFU, leflunomide; MTX, methotrexate; OD, once daily; RA, rheumatoid arthritis; RF, rheumatoid factor; RTX, Rituximab; Plts, platelets; PO, oral; SC, subcutaneous; SSZ, sulfasalazine; WCC, white cell count.

Two patients had intermittent neutropenia on prior DMARDs, potentially suggesting inherent patient susceptibility to drug-induced neutropenia. Indeed, the incidence of EON following rituximab may be underreported as neutrophils are infrequently checked after uncomplicated administration in routine clinical practice. If so, this could have implications for post-treatment infection risk management. Rituximab was otherwise well tolerated and demonstrated good clinical efficacy in all three patients.

The aetiology behind EON remains unclear. Late-onset neutropenia following rituximab is more common and has been well-documented, with several hypotheses proposed for its development. This includes the development of anti-neutrophil antibodies and abnormal B cell reconstitution following rituximab therapy and the amplification of T-granular lymphocyte populations that may go on to induce neutrophil apoptosis [5]. Studies have also reported a potential genetic cause for rituximab-induced neutropenia, with those carrying the high-affinity FcγRIIIa 158 V allele at greater risk. This allele also carries known susceptibility for RA [6], with homozygosity conferring both a more severe disease phenotype but also greater B cell depletion and thus greater depth of neutropenia post-rituximab [7]. However, EON does not occur reliably at first rituximab exposure in susceptible RA patients nor after every cycle, suggesting that other, potentially environmental, factors may also contribute.

We report the first dedicated case series of EON following rituximab in RA. We believe our findings highlight the need for increased awareness of EON and for recognition of the subsequent infection risk following rituximab, given the increased use in RA patients. Early monitoring of neutrophils in this context may provide additional insight and inform future risk stratification of this rare complication.

## Data Availability

The data underlying this article will be shared on reasonable request to the corresponding author.
